# Health education and factors influencing acceptance of and willingness to pay for influenza vaccination among older adults

**DOI:** 10.1186/s12877-015-0137-6

**Published:** 2015-10-26

**Authors:** Rawipun Worasathit, Wantanee Wattana, Kamolnetr Okanurak, Archin Songthap, Jittima Dhitavat, Punnee Pitisuttithum

**Affiliations:** 1 Faculty of Tropical Medicine, Mahidol University, 420/6 Ratchawithi Road, Ratchathewi, Bangkok, 10400 Thailand; 2 Bangkok Metropolitan Administration (BMA), Mitmaitree Road, Dindang, Bangkok, 10400 Thailand; 3 Sirindhorn College of Public Health, 89 Moo2 Thanon Trang-Kantang Thambon Kuanthani Amphoe Kantang, Trang Province 92000 Trang, Thailand

**Keywords:** Acceptance, Older adults, Health education, Influenza vaccination, Willingness to pay

## Abstract

**Background:**

The influenza vaccine is recommended in older population. However the immunization coverage varies globally. It has been reported as low as 10–20 % in some countries. This study explored the acceptance of and willingness to pay for influenza vaccination, comparing acceptance and willingness to pay before and after health education.

**Methods:**

The study was conducted with 2693 older people in Bangkok, Thailand. Participants were divided into an education group (*n* = 1402) and a control group (*n* = 1291). A validated questionnaire measuring acceptance of and willingness to pay for vaccination was administered during semi-structured interviews before and after education. Data on factors influencing acceptance were analyzed.

**Results:**

Participants’ mean age was 69.5 years, 80 % were women and 82.1 % had at least one co-morbidity. Of the participants, 43.5 % had previously received vaccination more than once, although 92.8 % expressed acceptance of vaccination. Acceptance was associated with a positive attitude toward vaccination (OR 2.1, 95 % CI 1.5–2.9) and a history of receiving vaccination (OR 4.1, 95 % CI 2.8–6.1). At baseline, there were no differences between the education and control groups in terms of work status (*p* = 0.457), co-morbidities (*p* = 0.07), medical status (*p* = 0.243), and previous vaccination (*p* = 0.62), except for educational background (*p* = 0.004). Acceptance of vaccination increased to 95.8 % (*p* < 0.001) after education and willingness to pay increased to 82.1 % (*p* < 0.001). Education significantly affected those with primary school-level education and no previous vaccination history, with acceptance increasing from 83.3 to 92.6 % (*p* < 0.001); more than twice as high as the control group (OR 2.4, 95 % CI 1.2–4.7). Viewing an educational video increased the proportion of participants with a high level of knowledge from 29.2 to 49.2 % (*p* < 0.001), and increased the proportion of participants with a positive attitude from 52.4 to 70.7 % (*p* <0.001). No significant difference was found in any parameter between the first and second assessment in the control group.

**Conclusions:**

The strategies to increase positive attitudes may enhance the acceptance of vaccination. Health education using an educational video demonstrated a significant impact on acceptance, willingness to pay, knowledge and attitude in older people. This may lead to increased sustainability of the immunization program in older people.

## Background

Influenza remains one of the more serious diseases affecting public health, and leads to increasing rates of morbidity, mortality, and hospitalization [1, 2]. Older people, especially those with chronic diseases, are the most high-risk population for severe illness and death [3–5]. The Centers for Disease Control and Prevention (CDC) estimated that during a regular flu season, 90 % of seasonal flu-related deaths in the US occurred in those aged 65 years and older [6]. Influenza infection also reduces functional independence, health and quality of life in older people, particularly those who suffer chronic diseases, and results in general economic loss [7].

The World Health Organization (WHO) and the CDC have recommended target groups for annual influenza vaccination including older adults [8]. In older adults, the vaccination reduces illness severity and complications by up to 60 % and deaths by 80 % [9]. A number of studies have confirmed the effectiveness and safety of the influenza vaccine. Influenza vaccination has reportedly prevented morbidity, hospitalization and mortality in older people with various conditions such as diabetes [10, 11], chronic heart disease [12] and patients with end-stage renal disease on hemodialysis [13]. Influenza vaccination is cost effective and safe for older adults [14, 15].

In addition, around the world, populations are aging. A number of countries are experiencing challenges in dealing with an aging population. Thailand’s population is also aging, with 11 % of the 64.5 million Thai citizens expected to be part of an aged society in 2020 [16]. Older people, particularly those with chronic diseases, benefit from protection against diseases through vaccination. Influenza and herpes zoster are the most common communicable diseases in older adults. If a large number of older adults are not vaccinated against influenza, the disease is likely to be more severe and result in death in more cases. If a large proportion of the older adult population receives influenza vaccination, dissemination of the disease within the community will be reduced and individuals will be protected against influenza infection.

In Thailand, influenza vaccination is provided free of charge to people aged 65 years or older and those with chronic diseases including diabetes, cerebrovascular disease, chronic kidney disease, asthma, chronic obstructive pulmonary disease, heart disease and those with cancer receiving chemotherapy. The EU Council of Ministers declared in 2009 to increase coverage of older age groups to a 75 % vaccination coverage by the 2014–2015 season [17]. In Thailand influenza vaccination coverage among those aged 65 years and older (both general population and high-risk population) increased gradually from 11.5 % in 2010, 14.3 % in 2011, to 19.6 % in 2012 [18], this vaccination coverage is relatively low in Thailand. Although some studies reported the cost-effectiveness of influenza vaccination [19–22], many of these studies did not investigate knowledge about vaccination and attitude towards vaccination, and did not study the factors associated with acceptance of and willingness to pay for vaccination among older Thai people (target vaccination recipients).

International studies have shown that acceptance of influenza vaccination is influenced to different degrees by factors such as age [23–26], sex [24], marital status [24, 27] and education level [23, 26], with factors varying in different regions and different racial/ethnic populations [28].

A study conducted with high-risk patients in the US comparing education and lottery incentives as ways to increase vaccination coverage found that an educational brochure encouraged people to get vaccinated against influenza [29]. Other studies have shown that health education using a mailed letter or a personal phone call successfully improved vaccination acceptance among various population groups, including older people, pregnant women and other high risk populations. However, most available studies were conducted in developed countries such as the USA, UK and Spain, where the health care systems, lifestyles and levels of knowledge in older people about vaccination differ from Thailand. Moreover, a survey of older Thai people conducted by the Thai National Statistical Office (NSO) reported that older Thais preferred receiving information by watching or listening rather than reading or via the internet [30]. As a result, education tools such as leaflets, surface mail, and text messages may not be suitable for older Thai people, particularly those who have impaired eyesight or who are less interested in reading [31–37].

The present study was conducted to explore acceptance of and willingness to pay for influenza vaccination among older adults, and to compare acceptance of and willingness to pay for influenza vaccination before and after receiving health education.

## Methods

The study was approved by the Ethics Committee of the Faculty of Tropical Medicine, Mahidol University, and the Bangkok Metropolitan Administration Ethics Committee for Human Research (BMAEC). Participant was informed and consented before included in the study. The study was conducted with ambulatory older people who attended one of the 68 Health Centers administered by the Department of Health, Bangkok Metropolitan Administration (BMA). Based on demographic profile, the population selected in this study represents the general older population in Thailand [30]. Each center runs regular activities and meetings as part of their care system for the ambulatory older population.

### Participants

The study population comprised people aged 60 years or older who lived in Bangkok. Participants were recruited from all of the 68 Health Centers in Bangkok. Those willing to participate in the study, who were self-assisted and able to read and sign the consent form were included in the study. Those who were not able to complete the questionnaires or could not watch the educational video were excluded.

### Data collection

Data were collected between 5 June and 5 July 2013. Around 40–50 participants from each of the 68 health centers were sampled using a purposive sampling technique. Participants’ acceptance of vaccination and willingness to pay for vaccination were assessed using validated questionnaires administered as semi-structured interviews at two points (before and after presentation of the educational video). Data from participants were kept confidential by the use of a participant code, meaning the participant’s name was not included on the questionnaires. Questionnaire content validity was verified by experts in the field of clinical science and social science (MD,PhD). Pre-testing was performed with a group of 30 elderly people, after which the questionnaire was modified and approved by the Ethics Committee of the Faculty of Tropical Medicine, Mahidol University, and the Bangkok Metropolitan Administration Ethics Committee for Human Research (BMAEC). The questionnaire consisted of: 1) basic sociodemographic characteristics such as age, sex, educational background, work status, monthly income, co-morbidities, medical status and history of previous flu vaccination; 2) knowledge assessment; 3) attitude assessment; and, 4) acceptance of and willingness to pay for vaccination.

Knowledge was assessed with 12 true or false questions as shown in Table 2 concerning the cause of influenza, high-risk groups and possible complications. To determine vaccination-related knowledge, the questions included the types of vaccine used in Thailand and the reasons for vaccination. Each item was given a score of 0 or 1. Knowledge scores were classified into three groups using Bloom’s Theory, which categorizes by percentage after the score is summed: ≤60 % represented low knowledge, >60–80 % moderate knowledge, and >80 % a high level of knowledge.

Attitude was assessed on a five-point Likert scale comprising 15 issues which were listed in Table 5. Questions about influenza infection included the importance and severity of the disease as well as medication. Vaccination-related items included the population groups that should be vaccinated, vaccine safety, vaccine effectiveness and optimal vaccination schedule. Scores of attitude were interpreted as follows. For the positive answers, Strongly agree, Agree, Neither agree nor disagree, Disagree, and Strongly disagree would receive scores of 5,4,3,2,and 1, respectively. For the negative answers, Strongly agree, Agree, Neither agree nor disagree, Disagree, and Strongly disagree would receive scores of 1,2,3,4, and 5, respectively. Mean attitude scores were classified into three groups by Best’s Theory: 1.00–2.33 = negative, 2.34–3.67 = neutral and 3.68–5.00 = positive.

Participants were divided into two groups: an education group and a control group. Participants enrolled at Health centers with an even number were assigned to the education group, while those enrolled at Health centers with an odd number were put in the control group. Participants in the education group were given a 1-h break after watching an educational video, after which the second assessment was performed. Participants in the control group did not watch the educational video, but were given a 1-h break after which the second assessment was performed.

### Intervention

The present study used a video developed by researchers as the educational tool. The video was based on health belief models describing people as taking action to prevent disease [33]. The content included influenza topics (i.e., influenza infection, seriousness, complications, transmission, influenza prevention and practice after contracting influenza) and influenza vaccination (i.e., effectiveness, safety, possible side effects, influenza vaccination program in Thailand and practices after receiving vaccination). To ensure participants could clearly understand the message, each frame of the educational video contained images as well as written information [33]. The video lasted for 10 min. The same group of experts in the field of clinical science and social science (MD, PhD) verified the accuracy of the video content. Pre-testing was performed with a group of elderly people, after which the video was modified and approved by the Ethics Committee of the Faculty of Tropical Medicine, Mahidol University and the BMAEC.

### Statistical analyses

The data were analyzed using SPSS Version 20 for Windows (IBM Corp., Armonk, NY). Descriptive statistics were used to describe basic sociodemographic characteristics. Knowledge and attitude were represented by the percentage of participants at each level and overall mean and median. Acceptance of and willingness to pay were represented by percentages. Chi-square analysis and then logistic regression were used to determine the associations between potential factors and acceptance or willingness to pay. The Mann–Whitney *U* test was used to compare mean attitude scores between those who accepted and did not accept vaccination due to non-normal distribution. The McNemar test was used to compare proportion data before and after video education. The Wilcoxon matched-pairs signed-ranks test was used to compare knowledge and attitude scores before and after according to non-normal distribution. All significance levels were set at 0.05.

## Results

A total of 3026 older adults were interested in the study, and 2693 (89 %) were included in the analysis. The mean age was 69.5 years (range 60–91 years). Around 55 % were aged 65–74 years, 24 % were aged 60–64 years, and 21 % were ≥75 years. The majority of participants were female (80 %), half were married and around 94 % were Buddhist. Nearly 80 % of participants were retired. In terms of educational background, 54.5 % had primary school-level education or less, 34.9 % were educated to secondary/diploma level and 10.6 % had a Bachelor’s degree or higher. About 62 % of participants had a monthly income of ≤5000 baht (≤154 USD).

At least one co-morbidity (underlying disease) was present in 82.1 % of participants, with hypertension being the most common (52.9 %), followed by dyslipidemia (40.9 %), diabetes (21.8 %) and heart disease (8.3 %). Most of the participants (90.4 %) had visited a medical facility within the past 1 year, and 78.7 % had received influenza vaccination advice from medical personnel. Around 76.8 % had a history of ever having received influenza vaccination (Table 1).

**Table 1:** Sociodemographic characteristics of participants

Characteristics	n (%)
Age (years) (*n* = 2693), mean 69.5 (5.95), range 60–91	
60–64	650 (24.1)
65–74	1488 (55.3)
≥ 75	555 (20.6)
Work Status (*n* = 2639)	
Out of work	2085 (79.0)
Still working	554 (21.0)
Education Level (*n* = 2688)	
≤ Primary level	1465 (54.5)
Secondary education/diploma	937 (34.9)
≥ Bachelor degree	286 (10.6)
Income (THB) (*n* = 2523) mean 7141.36 median 5000	
≤ 5000	1559 (61.8)
> 5000–10,000	511 (20.3)
> 10,000	453 (18.1)
Co-mordibity (*n* = 2686)	
No	480 (17.9)
Yes	2206 (82.1)
Hypertension	1420 (52.9)
Dyslipidemia	1099 (40.9)
Heart disease	224 (8.3)
Diabetes Mellitus	585 (21.8)
Other	251 (9.3)
Having a medical visit within the past 1 year (*n* = 2675)	
No	256 (9.6)
Yes	2419 (90.4)
Receiving influenza vaccination advice from medical personnel (*n* = 2591)	
No	553 (21.3)
Yes	2038 (78.7)
History of having received influenza vaccination (*n* = 2680)	
No	623 (23.2)
Yes	2057 (76.8)
Number of influenza vaccinations received (*n* = 1834 of 2457)	
One	765 (31.1)
Two	553 (22.5)
≥ Three	516 (21.0)

### Sources of information on influenza infection and vaccination

In total, 77 % of participants had received information on influenza infection. The most common information source was TV programs (58.9 %), followed by medical personnel (55.3 %), radio (20.6 %), newspaper (19.9 %), advertisement/leaflet (19.0 %) and friends (14.3 %).

Of the participants, 83.1 % had previously received information on influenza vaccination. The most common information source was medical personnel (61.0 %), followed by TV programs (51.4 %), radio (18.6 %), advertisement/leaflet (17.2 %), newspaper (17.1 %) and friends (13.4 %).

### Knowledge and attitude toward influenza infection and vaccination

Only 30 % of participants had a high level of knowledge about influenza infection, 38.7 % had a moderate level of knowledge, and 31.1 % had a low level of knowledge. The mean knowledge score was 8.4 (1.85) and the median was 8 (IQR 7–10). Participants tended to know about influenza transmission, practice after getting influenza, people who were in the high risk group and influenza complications, with more than 80 % of participants correctly answering these questions. However, participants knew little about the influenza organism (37.8 % correct) and symptoms (29.2 % correct). In terms of the influenza vaccination, not as many participants knew about influenza vaccination prevention (66.1 %), even though they knew that people should get a flu shot every year (96.7 %) (Table 2).

**Table 2:** Knowledge of influenza and vaccine

Questions	Corrected Answer n (%)
1. Influenza illness is caused by bacteria.	1019 (37.8)
2. Influenza contact is caused by sharing stuffs.	2240 (83.2)
3. Influenza symptoms are low fever, runny nose, nasal congestion, and headache.	785 (29.2)
4. The elderly who get influenza should take a cold shower to refresh themselves.	2482 (92.4)
5. The elderly who get influenza should work or do more exercises.	2099 (78.0)
6. When you have a high fever, you would rush to buy drugs from a drugstore.	2418 (90.1)
7. The elderly are at high risk of influenza complication.	2570 (95.5)
8. Conjunctivitis is a serious complication from influenza.	1417 (52.9)
9. Pneumonia is a serious complication from influenza.	2195 (81.8)
10. Influenza vaccine prevents only 2009H1N1 influenza.	1027 (38.3)
11. Influenza vaccine prevents only common cold.	1767 (66.1)
12. The reason why people should get flu shots yearly is the change in influenza strain.	2598 (96.7)
Mean score (sd)	8.40 (1.85)
Median score (IQR)	8 (7–10)

Nearly 55 % of participants had a positive attitude toward influenza and vaccination, while the remaining 45 % had negative or neutral attitudes. The mean of the total attitude score was 3.74 (0.36) and the median was 3.73 (3.53–4.00). Most participants were concerned about complications from influenza, and believed influenza is preventable and that there should be health education for high risk people. However, a number of participants disagreed with the impacts on health after contracting influenza and being vaccinated against influenza every year. Lots of participants had misconceptions about taking antibiotics. Participants felt that there were benefits of influenza vaccination and that vaccination was effective, but were curious about vaccination safety.

### Acceptance and willingness to pay for influenza vaccination

Most participants indicated they would like to receive free influenza vaccination (92.8 %), while 75 % indicated that vaccination would be acceptable when it incurred a cost. The number of people who indicated they needed vaccination decreased if the vaccination cost was higher. Around 37 % of participants could accept vaccination if it cost <100 baht (<3.08 USD); 29.4 % could accept if it cost 100–300 baht (3.08–9.23 USD); 6.2 % could accept if it cost 301–500 baht (9.26–15.38 USD); but only 0.5 % could accept if it cost >500 baht (>15.42 USD).

### Factors associated with influenza vaccination acceptance

It was found that nine factors associated with acceptance of an influenza vaccine among older adults. Acceptance of an influenza vaccine increased with age (*p* = 0.005). There were the relationships between acceptability of an influenza vaccine and work status (*p* = 0.014), having disease (*p* = 0.001), having medical visit within the past 1 year (*p* = 0.003), receiving influenza vaccination advice from medical personnel (*p* < 0.001), history of having received influenza vaccination (*p* < 0.001), received influenza information (*p* < 0.008), received influenza vaccination information (*p* < 0.001) and being positive attitude (*p* < 0.001).

Logistic regression analysis was used to determine the association between influenza vaccination acceptance and selected potential variables. A positive attitude and having a history of having received influenza vaccination were associated with influenza vaccination acceptance. Those who had a positive attitude were more likely to indicate that free influenza vaccination was acceptable than those with a negative or neutral attitude (OR 2.06, 95 % CI 1.49–2.85). Those who had a history of having received influenza vaccination were more likely to indicate that free influenza vaccination was acceptable than those with no vaccination history (OR 4.14, 95 % CI 2.81–6.10) (Table 3).

**Table 3:** Factors associated with acceptance of influenza vaccine among older adults (Logistic Regression)^a^

Factor	Acceptance of free influenza vaccination
	Adjusted odds ratio (95 % CI)
Age	
60–64	Reference
65–74	1.04 (0.72–1.50)
≥ 75	1.25 (0.76–2.08)
Work Status	
Out of work	Reference
Still working	0.81 (0.56–1.16)
Co-morbidity	
No	Reference
Yes	1.27 (0.84–1.92)
Having medical visit within the past 1 year	
No	Reference
Yes	1.01 (0.61–1.66)
Receiving influenza vaccination advice from medical personnel	
No	Reference
Yes	1.03 (0.69–1.54)
History of having received influenza vaccination	
No	Reference
Yes	4.14 (2.81–6.10)
Received influenza information	
No	Reference
Yes	0.93 (0.59–1.48)
Received influenza vaccination information	
No	Reference
Yes	1.19 (0.72–1.96)
Attitude Level	
Negative/Neutral	Reference
Positive	2.06 (1.49–2.85)

^a^Logistic regression analysis with odds ratio results

Mann–Whitney U tests were conducted to determine the difference between each attitude issue score and influenza vaccination acceptance. Table 4 shows that those who accepted free vaccination perceived influenza as an important disease in older adults more than those who did not accept free vaccination. Those who accepted free vaccination were also more likely to think that there is an impact on health after an older person becomes sick from influenza infection. Attitude issues about influenza vaccination related to acceptance were perception of influenza prevention, necessity of vaccinating every year, effectiveness or benefits and vaccination safety/low risk of an adverse reaction.

**Table 4:** Attitude comparison between those who accepted and did not accept influenza vaccination

Perceived Issue	Vaccination not accepted		Vaccination accepted		P value^*^
	mean	SD	mean	SD	
1. Influenza is not an important disease in the elderly.	2.99	0.107	3.35	0.029	**<0.001**
2. The elderly could develop serious complications from influenza infection.	4.09	0.075	4.21	0.019	0.183
3. Influenza infection in elderly people with chronic disease can lead to death.	4.07	0.073	4.18	0.018	0.147
4. There is no impact on health after an elderly person becomes sick from influenza infection.	3.31	0.096	3.55	0.025	**0.005**
5. Influenza is a preventable disease.	4.34	0.045	4.32	0.013	0.828
6. Antibiotics are needed in influenza infection.	2.10	0.079	2.15	0.023	0.956
7. One should give health education to the high risk elderly.	4.40	0.048	4.43	0.014	0.488
8. Influenza vaccination can prevent influenza infection.	4.27	0.054	4.47	0.013	**<0.001**
9. The elderly do not have to receive influenza vaccination.	3.55	0.083	3.92	0.022	**<0.001**
10. One does not have to be vaccinated against influenza every year.	3.09	0.091	3.67	0.024	**<0.001**
11. One should receive influenza vaccination before the rainy season.	3.91	0.077	3.94	0.020	0.934
12. Influenza vaccination is effective in preventing influenza infection.	3.88	0.074	4.10	0.018	**0.001**
13. Serious illness from influenza infection could be reduced by influenza vaccination.	4.01	0.067	4.12	0.019	**0.003**
14. Influenza vaccination is not safe, and can cause serious adverse reactions.	3.13	0.083	3.61	0.022	**<0.001**
15. Influenza vaccination is expensive.	2.43	0.079	2.43	0.024	0.336

*Significance determined using the Mann–Whitney *U* test

The bold numbers emphasized the significance of theses parameters toward vaccine acceptance

### Factors associated with willingness to pay for influenza vaccine among older adults who met free vaccination criteria

The six factors were significantly associated with willingness to pay for influenza vaccine among older adults who met free vaccination criteria from logistic regression model (Table 5). The model included potential variables from chi-square analysis which were marital status (*p* = 0.039), education level (*p* < 0.001), monthly income (*p* < 0.001), having medical visit within the past 1 year (*p* < 0.001), receiving vaccination advice from medical personel (*p* < 0.001), history of having received influenza vaccination (*p* < 0.001), receiving influenza information (*p* < 0.001), receiving influenza vaccination information (*p* < 0.001), knowledge level (*p* = 0.022) and attitude level (*p* < 0.001). Table 5 reveals that older adults who had secondary education/diploma, monthly income >10,000- < 30,000 Baht, received vaccination advice from medical personal, history of having received influenza vaccination, received influenza information, positive attitude were more likely to express their willingness to pay for influenza vaccine.

**Table 5:** Factors associated with willingness to pay for influenza vaccine among older adults who met and did not met free vaccination criteria (Logistic Regression)^a^

Factors	Willingness to pay among those who met free vaccination criteria	Factors	Willingness to pay among those who did not meet free vaccination criteria
	Adjusted odds ratio (95 % CI)		Adjusted odds ratio (95 % CI)
Marital status		Gender	
Married	Reference	Female	Reference
Single/Widow	0.83 (0.67–1.04)	Male	2.68 (1.24–5.79)
Education levels		Income	
≤ Primary school	Reference	≤5000	Reference
Secondary school/diploma	1.58 (1.22–2.03)	>5000-10,000	1.06 (0.63–1.79)
≥ Bachelor	1.24 (0.80–1.92)	>10,000- < 30,000	3.47 (1.44–8.37)
Income		≥30,000	0.74 (0.22–2.47)
≤ 5000	Reference	Receiving influenza vaccination advice from medical personnel	
> 5000–10,000	1.23 (0.92–1.65)	No	Reference
> 10,000–<30,000	1.68 (1.12–2.51)	Yes	1.07 (0.61–1.87)
≥ 30,000	1.10 (0.59–2.04)	History of having received influenza vaccination	
Having medical visit within the past 1 year		No	Reference
No	Reference	Yes	2.64 (1.53–4.55)
Yes	1.41 (0.99–2.02)		
Receiving influenza vaccination advice from medical personnel		Receiving influenza information	
No	Reference	No	Reference
Yes	1.46 (1.07–1.97)	Yes	1.44 (0.73–2.83)
History of having received influenza vaccine		Receiving influenza vaccination information	
No	Reference	No	Reference
Yes	1.35 (1.00–1.81)	Yes	1.60 (0.76–3.40)
Receiving influenza information		Attitude Levels	
No	Reference	Negative/Neutral	Reference
Yes	1.48 (1.10–2.00)	Positive	1.72 (1.08–2.75)
Receiving influenza vaccination information			
No	Reference		
Yes	1.05 (0.74–1.48)		
Knowledge level			
Low/Moderate	Reference		
High	0.96 (0.75–1.24)		
Attitude levels			
Negative/Neutral	Reference		
Positive	1.53 (1.23–1.90)		

^a^Logistic regression analysis with odds ratio results

### Factors associated with willingness to pay for influenza vaccine among older adults who did not meet free vaccination criteria

The four factors significantly associated with willingness to pay for influenza vaccine among older adults who did not meet free vaccination criteria from logistic regression model (Table 5). The model included potential variables from chi-square analysis which were gender (*p* = 0.012), income (*p* = 0.004), receiving vaccination advice from medical personnel (*p* = 0.003), history of having received influenza vaccination (*p* < 0.001), receiving influenza information (*p* < 0.001), receiving influenza vaccination information (*p* < 0.001) and attitude level (*p* = 0.004). Table 5 reveals that older adults who were males, had monthly income >10,000- < 30,000 Baht, history of having received influenza vaccination, and positive attitude were more likely to express their willingness to pay for influenza vaccine.

### Characteristics of education and control group participants

There were 1402 participants in the education group and 1291 in the control group. Baseline characteristics for both groups were no different in terms of their stratified age proportion (*p* = 0.115), work status (*p* = 0.457), monthly income (*p* = 0.296), co-morbidity status (*p* = 0.07), having had a medical visit within the past one year (*p* = 0.243) and having a previous vaccination history (*p* = 0.62). However, overall, participants in the education group had a lower education level than those in the control group (*p* = 0.004). In the education group, 57.3 % had primary school education or less, 33.4 % had secondary/diploma level education and 9.3 % had a Bachelor’s degree or higher. In the control group, 51.5 % had primary school education or less, 36.4 % had secondary/diploma level education and 12.1 % had a Bachelor’s degree or higher.

### Impact of video education on acceptance

In the education group, there was a significant increase in the acceptance of free influenza vaccination after video education from 91.4 % to 95.8 (*p* < 0.001). The increase in acceptance in those with primary school-level education was also a significant (from 91.3 to 95.8 %, *p* < 0.001). The control group showed no change in acceptance between the first and second assessment: 94.4 % vs 94.8 % (*p* = 0.568) for the whole group and 93.4 % vs 93.2 % (*p* = 1.00) in those with primary school-level education (Table 6).

**Table 6:** Acceptance of influenza vaccination in the elderly before and after video education

Issues	Education group			Control group		
	Before n (%)	After n (%)	P value*	Before n (%)	After n (%)	P value*
All elderly	1281 (91.4)	1343 (95.8)	<0.001	1219 (94.4)	1224 (94.8)	0.568
*n* = 2693; education = 1402,control = 1291						
Acceptance of a free influenza vaccination.						
Elderly with primary school-level education	739 (91.3)	768 (95.8)	<0.001	619 (93.4)	618 (93.2)	1
*n* = 1465; education = 802,control = 663						
Acceptance of a free influenza vaccination.						
Elderly with primary school-level education and no history of receiving influenza vaccine	170 (83.3)	189 (92.6)	0.002	148 (84.1)	148 (84.1)	1
*n* = 388; education = 204,control–176						
Acceptance of a free influenza vaccination.						

^*^Significance determined using the McNemar test

When a subset of participants with primary school-level education and no influenza vaccination history was analyzed, the impact of the educational video was obvious (education group: 83.3 % vs 92.6 %, *p* = 0.002; control group: 84.1 % vs 84.1 %; *p* = 1.00) (Table 6). Chi-square tests found no difference between the groups at baseline (83.3 % vs 84.1 %, *p* = 0.842), but significantly higher acceptance in the education group compared with the control group at the second assessment (92.6 % vs 84.1 %, *p* = 0.009). In addition, the educational video had more than twice the effect on acceptance (sex and co-morbidity adjusted OR 2.42, 95 % CI 1.24–4.71).

### Impact of video education on willingness to pay

In the education group, video education increased willingness to pay for influenza vaccination from 72.2 to 82.1 %; an increase of 9.9 % accounting for a 13.7 % increase (*p* < 0.001). The number of participants who expressed willingness to pay decreased as vaccination cost increased. After the educational video, the number of participants willing to pay increased from 36.9 to 43.2 % when vaccination costs were <100 baht (<3.08 USD), and from 27.6 to 30.6 % when vaccination costs were 100–300 baht (3.08-9.23 USD). In the education group, there was a significant increase in willingness to pay in those with primary school-level education (67.4 to 80.1 %; increase of 12.7 % accounting for an 18.8 % increase, *p* < 0.001). The control group showed no difference in willingness to pay between the first and the second assessment: 77.9 % vs 79.4 % (*p* = 0.121) in all control group participants and 72.1 % vs 74.7 % (*p* = 0.094) in those with primary school-level education.

When a subset of participants with primary school-level education and no influenza vaccination history was analyzed, the impact of the educational video was significant (education group: 56.2 % vs 69.6 %, *p* = <0.001; control group: 60.8 % vs 60.9 %, *p* = 1.00). Chi-square tests found no difference between the groups at baseline assessment (56.2 % vs 60.8 %, *p* = 0.364), and at the second assessment (69.6 % vs 60.9 %, *p* = 0.076).

### Impact of video education on knowledge

Before video education, 29 % of those in the education group had a high level of knowledge, 39.1 % had a moderate level and 31.7 % had low level. After video education, a higher proportion of participants reported a high level of knowledge (from 29.2 to 49.2 %, accounting for a 68.5 % increase, *p* < 0.001). The control group showed no change in the proportion of participants who had a high level of knowledge between the first and second assessment (31.4 % vs 33.2 %, *p* = 0.134) (Table 7). In terms of knowledge level, the mean score of the education group increased from 8.38 to 9.26 and the median (IQR) increased significantly from 8 (7–10) to 9 (8–11), (*p* < 0.001). The mean score of the control group leveled off at 8.52 in the second assessment.

**Table 7:** Knowledge and attitude about influenza infection and influenza vaccination before and after video education

Levels	Education group			Control group		
	Before n (%)	After n (%)	P value*	Before n (%)	After n (%)	P value*
Knowledge Levels						
Low/Moderate	993 (70.8)	712 (50.8)		885 (68.6)	863 (66.8)	
High	409 (29.2)	690 (49.2)	<0.001	406 (31.4)	428 (33.2)	0.134
Attitude Levels						
Negative/Neutral	667 (47.6)	411 (29.3)		551 (42.7)	536 (41.5)	
Positive	735 (52.4)	991 (70.7)	<0.001	740 (57.3)	755 (58.5)	0.474

^*^Significance determined using the McNemar test

### Impact of video education on attitude

At baseline, 52.4 % of participants in the education group had a positive attitude and 47.5 % were neutral. After video education, the proportion of participants who had positive attitude rose significantly from 52.4 to 70.7 %, accounting for a 34.9 % increase (*p* < 0.001) (Table 7). In the control group, nearly the same percentage of participants had a positive attitude in the first and second assessment (57.3 % vs 58.5 %).

Video education enhanced the attitude of participants, with the mean score increasing from 3.72 to 3.86 and the median (IQR) increasing significantly from 3.73 (3.47–3.93) to 3.87 (3.63–4.07) (*p* <0.001). The mean attitude scores increased significantly after video education for 11 out of 15 issues. The view that antibiotics are unnecessary in influenza infection changed from negative to neutral after education. In addition, there was a change from neutral to positive relating to the impact on health after an older person becomes sick from influenza infection and if a vaccination should be received every year. However, without exposure to the educational video, the mean score for the control group leveled off between the first and second assessment (3.76 vs 3.77), and there was no change in attitude level on any issue.

## Discussion

The present study was performed with a large sample size, which increased the reliability of the results. Characteristics of older participants in our study were consistent with Annual Report Situation of elderly Thais in terms of co-morbidities (DM 21.8 % from this study and 13.3 % from Annual report, heart disease 8.3 and 7 %, cerebrovascular disease 2.6 and 1.6 %), monthly income (7141 Baht and 7495 Baht), education level (primary school level 54.5 and 68.92 %), work status (still work 21.0 and 19.9 %), source of information (tv program 58.9 and 57.4 %, radio 20.6 and 32.8 %) [30, 38].

The percentage of participants who had heard of influenza infection in older adults in our study was similar to the findings of a study with people living along the Thai-Myanmar border; 76.6 % compared with 76.4 % [39]. In terms of influenza vaccination, 83.1 % of older people in our study had heard of influenza vaccination, while 94 % of those in a study with American older people had heard that it was recommended that they be vaccinated against influenza [28]. Our study also found that the most common source of information for older people about influenza infection was TV programs, followed by medical personnel, radio, newspaper, advertisement/leaflet and friends. The previous study with a population living along the Thai-Myanmar border found slightly different results in that a TV program was the most common, followed by radio, newspaper, health center, friends, and brochures/posters [39]. However, this supports our finding that TV programs are the most common source through which Thais received information on influenza infection. Although the most common source for information on influenza infection was TV programs, older people reported that the most common source for information on influenza vaccination was medical personnel. This suggests that influenza vaccination information disseminated through health centers may be limited and in general, may not be sufficient. Therefore, sources of influenza vaccination information should be expanded to include TV programs, radio, newspapers and advertisements/leaflets to promote influenza vaccination programs and immunization coverage.

Most of older adults in our study (69.8 %) had moderate or low level knowledge, a finding similar to that of an earlier study (2008) in a group of 20 older people in the Muang district, an urban community of Chiang Rai province where little was known about influenza infection and influenza vaccination [40]. In contrast, international studies have found that older people were knowledgeable about influenza causes, transmission, symptoms, complications, practices and vaccination [41, 42]. Our findings suggest that few older adults in Thailand have sufficient knowledge about influenza causes, symptoms, complications and vaccination. This indicates that current health education programs on influenza and vaccination may not be adequate.

Around half of older adults in our study had a positive attitude toward vaccination. They were concerned about the serious complications from influenza, but less concerned about impacts on health after becoming sick from influenza infection. Most had a misconception about taking antibiotics during influenza infection, a finding that differed from a 2006 study with Greek elders [41]. In addition, we found that older people less perceived getting an influenza vaccination every year and the safety of vaccination.

Even though 92.8 % of older people in our study were willing to receive a free influenza vaccination, only 76.8 % had history of having received influenza vaccination. Of these, 31.1 % had received vaccination once, 22.5 % had received vaccination twice, and 21.0 % had received vaccination three or more times (the influenza vaccination program in Thailand has been provided free of charge since 2008). This finding highlights the relatively low immunization coverage in Thailand [4, 18] and implies that annual influenza vaccination was not consistent for individuals.

Approximately 37 % of older people were willing to pay for influenza vaccine if the cost was below 100 baht (<3.08 USD), and 29.4 % would pay if the cost was between 100 and 300 baht (3.08-9.23 USD). Only 6.7 % could afford to pay for vaccination if the cost was more than 300 baht (>9.23 USD). However, in Thai hospitals, vaccination prices ranged from around 350 (10.77 USD) in public hospitals to 700 baht (21.54 USD) in private hospitals (not including medical charges). A study with Hong Kong Chinese people in 2004 found that 46 % felt HK$50 (6.39 USD) would be a reasonable cost for vaccination, and 32 % accepted a cost in the range of HK$50–$100 (6.39-12.77 USD) [43].

Our study indicates that having a positive attitude and a history of previous influenza vaccination were the main factors associated with influenza vaccination acceptance. As highlighted in other studies, people who had a positive attitude were more likely to accept influenza vaccination [44–46]. In terms of previous vaccination history, our finding was consistent with previous studies that having a vaccination history was a powerful indication of vaccination acceptance [42–44, 47–51]. In addition, older people who accepted free vaccination felt that influenza is an important disease in older adults [45, 47, 49, 52–54], and that there is an impact on health after becoming sick from influenza [55–57]. Attitude issues about influenza vaccination related to acceptance were perception of its effectiveness/benefits [25, 27, 41, 50, 51, 53, 55, 56, 58] and perception of its safety/low risk adverse reaction [25, 27, 48–51, 55, 57–59].

Promoting a positive attitude toward influenza infection and influenza vaccination is a potentially effective method of increasing vaccination acceptance and immunization coverage. Moreover, some studies have found that elderly people who were vaccinated against seasonal influenza were more likely to receive pandemic influenza vaccination [44, 60].

Other sociodemographic characteristics found to be significantly related to influenza vaccination included age [23–25], marital status [24, 27], education level [23, 26], income [23], having co-morbidities [23, 43] and influence of healthcare personnel [15, 24, 27, 43, 46, 50, 51, 58, 59]. These characteristics varied in different countries, races, ethnicities and cultures. Improvements in technology and global travel [61] mean that areas tend to have mixed cultures; for example, by 2030 in the US, Blacks Hispanic and Asian older adults will comprise a significantly larger proportion than they did in the past. The US census also estimates that by 2050, the older population in the US will look different. It has been predicted that within 10 to 20 years, ethnic minorities will be the new majorities, and in essence, Asians will have the highest rates of growth numerically [62]. The United States encounter the continuous influx of refugees from Southeast Asia since 1975. They reported that Asian Pacific Americans remain one of the most poorly understood minorities [63] and there are smaller studies indicating these Asian population groups [64]. Thus, understanding of this population is needed for health policy maker and service providers in order to provide facilities against health problems and reduce health care costs of the developed nation [63]. Moreover, in an effort to control contagious and highly infectious disease like influenza, global cooperation is needed. Each area should investigate and report population characteristics, knowledge, attitude, vaccination acceptance and influential factors to focus vaccination and improve immunization coverage throughout the world, meaning global prevention may be within the realm of possibility.

This study evaluated the impact of health education on acceptance and willingness to pay for influenza vaccination by assessing key factors before receiving education (baseline) and after receiving education according to principles of health education assessment [33]. The education tool used correlated with the principles of health education for the elderly [33]. We were able to examine the direct impact of the success of education (intervention), and our results showed that education using a video had a significant impact. Our study also assessed acceptance of and willingness to pay in the control group at two time points and the results showed no difference, indicating that the effect of education was true effect and there was less intervening effect that could distort our conclusions.

Our study represents older people in a developing country and has shown that vaccination acceptance in older people increased from 91.4 to 95.8 % after watching the educational video. Similar studies in developed countries have been conducted with varying results. A study in Spain showed that the use of a printed letter on led to a 3.6 % increase in the immunization rate from 38.1 to 41.7 % [36]. A UK study found an increase in vaccination uptake in older people from 46.7 to 67.9 % after they received a personal letter of invitation to attend an influenza vaccination clinic [31].

The impact of the educational video was significant in older people who had primary school-level education or less and who had no previous history of influenza vaccination. It is noteworthy that this subset initially had lower vaccination acceptance than the overall study population. After video education, overall vaccination acceptance increased from 83.3 to 92.6 %. Moreover, logistic regression showed the video education increased acceptance by more than two times. Our results highlighted that video education also had benefit for the group of older Thai people who were less likely to accept influenza vaccination. A US study (1996) found that an educational brochure had a striking effect on influenza immunization among high-risk patients who did not accept immunization the prior year [29]. Health education may be important among a number of countries where its population education is relatively low or similar to Thailand data (less than 50 % of population attain secondary education) such as Mexico, Brazil, South Africa, India, China, Indonesia, Philippines, and Vietnam [65].

Our study showed the importance of using a different form of health education (educational video). Other studies in developed countries have used other education forms such as a letter [31, 34, 36] or brochure [29], which might not have been suitable for older people due to eyesight impairment or less interest in reading long or complex statements [33].

The UK study also showed that health checks by a practice nurse who offered influenza vaccination resulted in an immunization rate increase from 48.7 to 74.3 %. However, this intervention was costly and its effect on influenza vaccination rates was modest [31]. Another study focused on hard-to-reach populations such as drug abusers, sex workers, immigrants, homeless people and older people, using a multilevel community-based method that included cooperation among neighbors, local community staff, nurses and physicians. After intervention, interest in receiving vaccination increased from 80 to 94 %. Although the multilevel community-based method had an effective outcome, the intervention required collaboration from many institutes, meaning a larger budget and a longer time were required [32].

Our study also measured willingness to pay for vaccination, a factor that has not been considered in previous studies. Our results showed the educational video had a high impact on willingness to pay (a 13.7 % increase overall, and an 18.8 % increase in older people with primary school-level education). This increase may result from increased recognition of the benefit of influenza vaccination [33], and implies that vaccination recipients may be willing to pay when more expensive new generation vaccines become available, although these are relatively more expensive for the government. Although the chi-square test showed no difference in willingness to pay between the education and control groups at the second assessment, other economic factors may be related to willingness to pay such as income level; if the vaccination is more expensive, fewer older people would be willing to pay. Less than a half of older adults in our study could afford vaccination if the price was less than 100 baht (<3.08 USD), but the market price has been much higher. However, the percentage of older people in the education group who were willing to pay for vaccination after the education video increased from 56.2 to 69.6 %.

The present study included participants aged ≥60 years. Retirement is an option for Thai people at the age of 60 years. While vaccination acceptance increased after education, our results showed that affordability is a barrier to vaccination. This indicates that it is important for the Thai government to reconsider the influenza vaccination program criteria to cover older people aged ≥ 60 years.

Aside from acceptance of and willingness to pay for influenza vaccination, the educational video improved the knowledge and attitudes of older people. This is important as Thailand is a developing country with limited knowledge, negative attitude and low influenza vaccination coverage, and differs from developed countries [41, 42]. Our finding that education had an effect on knowledge and attitudes about influenza vaccination was similar to a study with hematopoietic stem cell transplantation patients [66]. Our study found that those who accepted vaccination felt that becoming sick from influenza infection would have an impact on health, and that people should be vaccinated against influenza every year. After video education, there was an increase in positive attitudes toward these two issues. Other attitude issues related to vaccination acceptance also showed an increase in mean scores after education, including perception of vaccination preventing influenza and the effectiveness/benefit of vaccination. Improvement in knowledge and attitude resulted in increased awareness of influenza prevention, which in turn may lead to increased immunization coverage and sustainability of the program [67, 68].

Our quasi-experimental study suggested that education is a successful method of improving vaccination acceptance and willingness to pay for vaccination among elderly people in an aging society. Education intervention is necessary for implementation of vaccination programs, particularly in a developing country such as Thailand due to poor knowledge about vaccination, negative attitudes and low influenza vaccination coverage. An educational video is a helpful tool for health education for older people. It is simple and has both pictures and sound, which may help older people to pay attention, particularly if they have impaired eyesight and less concentration when reading [33]. This indicates that an educational video is an alternative educational tool for older adults that may lead to the sustainability of immunization programs.

### Study limitations

A limitation of our study is that the potential factors that showed correlation with acceptance of or willingness to pay in the cross-sectional part of the study do not allow causality to be identified due to the lack of temporal sequence between factors and outcomes.

## Conclusions

Influenza vaccination acceptance is associated with having a positive attitude. Therefore, strategies to improve attitudes toward vaccination should be implemented to improve immunization coverage. Furthermore, TV programs are the most common media for conveying information on influenza infection to older adults. However, access to information on influenza vaccination is currently restricted to health care centers. Consequently, sources of vaccination information should be expanded to include TV, radio, newspapers, and leaflets promoting the influenza vaccination program. Health education using an educational video demonstrated a significant impact on acceptance of and willingness to pay for influenza vaccination as well as improving knowledge and attitude in elderly Thais. Health education is particularly important if the vaccination is not available for free. In addition, educational videos provided by the government through tele-media/TV program during influenza vaccination campaigns may improve immunization coverage, particularly in older adults who have low vaccination acceptance. Increasing knowledge and positive attitudes about vaccination will lead to sustainability of the influenza immunization program in low to mid income older populations worldwide.

## Abbreviations

CDC: The Centers for Disease Control and Prevention
WHO: The World Health Organization
NSO: Thai National Statistical Office
BMAEC: The Bangkok Metropolitan Administration Ethics Committee for Human Research
BMA: Bangkok Metropolitan Administration
